# Immunoproteomic Lessons for Human Respiratory Syncytial Virus Vaccine Design

**DOI:** 10.3390/jcm8040486

**Published:** 2019-04-10

**Authors:** Daniel López, Alejandro Barriga, Elena Lorente, Carmen Mir

**Affiliations:** Centro Nacional de Microbiología, Instituto de Salud Carlos III, 28220 Majadahonda (Madrid), Spain; abarriga@isciii.es (A.B.); elorente@isciii.es (E.L.); c.mir@isciii.es (C.M.)

**Keywords:** antigen processing, immune response, HLA, immunoproteomics, mass spectrometry, respiratory infectious disease, T cells, vaccine

## Abstract

Accurate antiviral humoral and cellular immune responses require prior recognition of antigenic peptides presented by human leukocyte antigen (HLA) class I and II molecules on the surface of antigen-presenting cells. Both the helper and the cytotoxic immune responses are critical for the control and the clearance of human respiratory syncytial virus (HRSV) infection, which is a significant cause of morbidity and mortality in infected pediatric, immunocompromised and elderly populations. In this article we review the immunoproteomics studies which have defined the general antigen processing and presentation rules that determine both the immunoprevalence and the immunodominance of the cellular immune response to HRSV. Mass spectrometry and functional analyses have shown that the HLA class I and II cellular immune responses against HRSV are mainly focused on three viral proteins: fusion, matrix, and nucleoprotein. Thus, these studies have important implications for vaccine development against this virus, since a vaccine construct including these three relevant HRSV proteins could efficiently stimulate the major components of the adaptive immune system: humoral, helper, and cytotoxic effector immune responses.

## 1. Knowing the Enemy: The Human Respiratory Syncytial Virus

Human respiratory syncytial virus (HRSV) [1], a member of the *Pneumoviridae* family of the order *Mononegavirales*, is the major cause of severe lower respiratory tract illnesses, such as pneumonia and bronchiolitis, in newborns and young children [2,3,4]. Infection rates approach 70% in the first year of life [5] and virtually 100% by 2–3 years of age [6]. This enveloped Orthopneumovirus infects people of all ages, with frequent natural reinfections [7]. Although mild infections are generally reported in healthy adults, HRSV also poses a serious health risk for immunocompromised [8,9] or elderly individuals [10,11], in addition to the pediatric population, when HRSV exposure results in hospitalization, which occurs among approximately 2–3% of infected infants. Many times, the respiratory damage does not end with the resolution of the infection because some of these children will develop an increased risk for recurrent wheeze and asthma [12]. Worldwide, approximately 3.5 million hospital admissions are associated with HRSV medical complications each year, and the mortality is estimated at more than a quarter of a million deaths each year, mainly in developing countries [13,14]. In addition, HRSV is a major nosocomial hazard in hospital or healthcare service units for patients of all ages [15], involving an important medical as well as economic impact. 

After the first HRSV isolation from children with respiratory illness in 1957 [16], the NIH quickly initiated a program to develop a vaccine against this virus. The approach was the purification of HRSV particles and their inactivation with formaldehyde, the same methodology that had been productively applied previously to other similar enveloped viruses. The clinical trial of the mid-60s was a complete failure, because the vaccinated children did not become protected, since the naturally occurring HRSV infection was significantly higher in immunized kids than in control children [17]. Notably, this immunization greatly increased the rate of hospitalization from 5% in the control group to an astonishing 80% of formaldehyde-inactivated HRSV group individuals, and two fatalities occurred in this last group [17]. Initial postmortem examination showed peribronchiolar monocytic infiltration dominated with eosinophils involving extensive bronchopneumonia and patchy atelectasis with emphysema and pneumothorax in dead children [17]. However, subsequent studies of the autopsy samples showed the presence of lymphocytes, neutrophils, and macrophages in the peribronchial infiltrates and in the bronchial exudates [18]. These studies and others made later delineated the role of cellular immune responses to the disease associated with the formaldehyde-inactivated vaccine. This devastating trial has had a profound impact on HRSV vaccine development, and the efforts of following decades have been made using different experimental approaches (reviewed in [19]), including particle-, viral protein subunit-, vector-, and viral RNA or DNA-based candidates. Currently, to address the priority of all the organizations that recognize the unmet need for a vaccine (including the World Health Organization, the nonprofit global health organization (PATH), the Bill & Melinda Gates Foundation and the pharmaceutical industry), a protective vaccine that specifically promotes an antiviral immune response must be developed.

HRSV has a single-stranded, negative-sense RNA genome that encodes 11 proteins. Similar to all pneumoviruses, HRSV is sequentially transcribed by the viral RNA polymerase (L) into discrete mRNAs, with a transcription initiation at a single 3′ promoter. Therefore, this process involves a sequential start-stop-restart mechanism to produce the different viral mRNAs [1]. As the polymerase occasionally fails to reinitiate the downstream mRNA at each stop-restart junction, a loss of transcription of further downstream genes occurs [1,20]; hence, there is an mRNA synthesis gradient that is inversely proportional to the distance of the gene from the 3′ end of the viral genome. Thus, in HRSV, promoter-distal genes are expressed less efficiently than promoter-proximal genes [20,21].

In addition to the multiple layers of defense against viral infection, HRSV induces mucosal and systemic humoral and cellular responses. Specific secretory antibody responses correlating with the time required for virus clearance and lower titers of nasal IgA and serum IgG neutralizing antibodies against the F, but not the G, protein are associated in patients with increased rates of infection [22,23]. Normal antiviral immune response against HRSV is mainly Th1, while a Th2-biased CD4 response was observed using formaldehyde-inactivated HRSV vaccine. Studies of mouse models evaluating CD8^+^ and CD4^+^ T-lymphocyte subsets showed that both cytotoxic MHC class I- and helper MHC class II-restricted cellular responses are fundamental in clearing HRSV infections [24]. In addition, children with defective T-cell immune responses show both high viral titers and increased HRSV-mediated disease severity [25,26]. 

The human leukocyte antigen (HLA) class I and II antigen processing pathways are the key elements to trigger functional antiviral CD8^+^ and CD4^+^ T lymphocyte responses, respectively. Proteolytic degradation by cytosol proteases, mainly proteasomes, of the newly synthesized viral or cellular proteins, some of which synthesis or folding are defective generates 8–10 residues long peptides. These short peptides, after translocation to the endoplasmic reticulum (ER) lumen by transporters associated with antigen processing, bind to the newly synthesized HLA class I molecules. These stabilized peptide/HLA class I complexes are then exported to the surface of cells for cytolytic CD8^+^ T cell recognition [27]. Instead, antigen presenting cells synthesize HLA class II molecules that, after insertion in the ER, are later transported to endosomal compartments without binding antigenic peptides. Next, these organelles fuse with late endosomes, which can contain exogenous protein material as viral particles and/or extracellular host proteins that were previously engulfed by endocytosis, phagocytosis, or pinocytosis. These exogenous proteins can be processed by the different lysosome-resident cathepsins, yielding cellular and viral peptides of different lengths [28]. These peptides, up to 30 residues long, stabilize the HLA class II molecules and then, these HLA peptide/class II complexes are transported to the cell membrane where they are exposed for CD4^+^ T helper cell recognition [29]. In absence of appropriate HLA class I and II-restricted T cell recognition both cellular and humoral immune responses cannot be efficiently activated and thus, the infective virus could spread within the whole organism with fatal results for the host.

In this context, several immunoproteomics studies performed in our laboratory have identified the target viral proteins and the ligands and epitopes presented by several common human leukocyte antigen (HLA) class I and II alleles against which the cellular immune responses are focused during HRSV infection.

## 2. HLA Class I Immunoproteomics: The HRSV Transcription Groups Determine the Immunoprevalence of the HLA Class I Response

**Figure 1:**
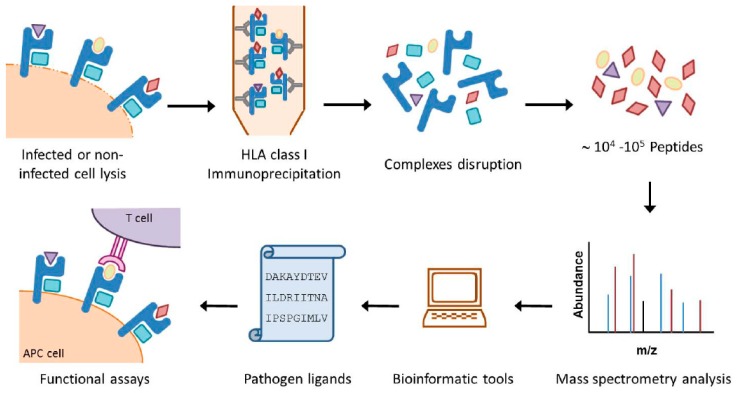
Overview of the HLA immunoproteomic approach. Both large amounts of pathogen-modified and healthy cells expressing the HLA class I or II molecules of interest are lysed in the presence of protease inhibitors. The HLA class I complexes are immunoprecipitated by affinity chromatography with specific anti-HLA mAbs and the HLA/peptide complexes are denatured. The resulting peptide mixtures recovered after an ultra-filtration step are separated by high performance liquid chromatography and analyzed by mass spectrometry. Bioinformatics tools are then used to obtain the sequences of the pathogen ligands. Finally, synthetic peptides, which can be used in functional analysis such as HLA/peptide stability assays, ELISPOT, intracellular cytokine staining assays or cytotoxicity assays, are generated.

Throughout this century, different HRSV epitopes restricted by several HLA class I molecules have been identified using cytotoxic T lymphocytes (CTL) from seropositive individuals (Table 1) [30,31,32,33,34]. However, these experiments were performed only with synthetic peptides of individual proteins and not against the complete virus and thus, only partial information could be obtained with the synthetic peptides analysis. In contrast, in 2006 using immunoprecipitation of HLA class I molecules and mass spectrometry analysis from HRSV-infected cells, Meiring et al. published a study that attempted to elucidate the nature of the natural virus ligands restricted by two common HLA class I molecules from HRSV A2 strain-infected peripheral blood mononuclear cells and dendritic cells [35]. In this in vitro analysis, using strong cation exchange fractionation and stable isotope tagging one viral ligand for each of the HLA-A*02 and HLA-B*07 class I molecules was identified [35]. The basic strategy for the identification of HLA class I ligands by immunoproteomics is summarized in Figure 1. However, an issue remained: is the HRSV immune response restricted by a single immunodominant viral ligand in both HLA class I alleles studied or, on the contrary, could each particular HLA molecule present different ligands of this small virus simultaneously? A second in vitro immunoproteomics study using sequential affinity and micro liquid chromatographies, and comparing the HLA ligands isolated from uninfected or HRSV Long strain-infected human HMy2.C1R cells transfected with HLA-B*27 demonstrated the existence of nine naturally processed HLA-B27 ligands from six viral proteins [36]. With this same methodology, two new immunoproteomics analyses infecting human lymphoblastoid cell lines with HRSV Long strain were carried out. The first analysis of HLA-A*02^+^, HLA-B*07^+^ HRSV-infected cells demonstrated that a total of five ligands were endogenously processed and presented by these HLA class I molecules in the same virus-infected cells: three ligands by HLA-A*02 and two by -B*07 [37]. Finally, immunoproteomics analyses of different HLA-C class I molecules identified a physiologically processed HLA ligand derived from HRSV matrix protein that used alternative interactions to the anchor motifs previously described in its presentation of the HLA-C*04 class I molecule [38]. Such analyses also identified a canonical natural ligand bound to the HLA-C*07 allele derived from the G protein [37].

In summary, using similar immunoproteomics approaches, three HLA-A*02, three HLA-B*07, nine HLA-B*27, and one of either HLA-C*04 or HLA-C*07 natural ligands have Figure been identified (Table 2), raising the total number to 17 HRSV HLA class I ligands that are derived from 9 of the 11 viral proteins encoded by the viral genome (Table 2), and show that each particular HLA molecule could present different HRSV ligands simultaneously. These five HLA class I molecules cover approximately 70% of the human population [40].

**Figure 2:**
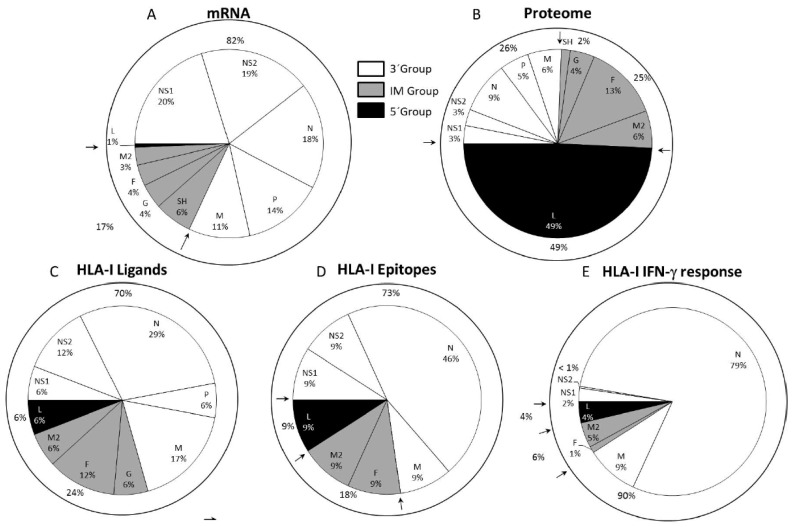
HLA class I ligands from HRSV identified by mass spectrometry and their relation to the viral mRNA, proteome, and T cell cytotoxic immune response. **A** panel: Pie chart representation of the HRSV genome indicating the relative abundance of mRNAs from the viral transcription, which was measured as the mRNA molar ratio percentage [21,41,42]. The abbreviations used for viral proteins were NS1 (non-structural protein 1), NS2 (non-structural protein 2], N (nucleoprotein), P (phosphoprotein), M (matrix protein), SH (small hydrophobic protein), G (glycoprotein), F (fusion protein), M2-2 (matrix protein 2], and L (polymerase), which were grouped into three different transcription groups: 3′ (white), intermediate (IM) (gray), and 5′(black) separated by arrows as in previously publications [21,41,42]. For each protein included into its respective transcription group, the proteome percentage is shown in **B** panel; the HLA class I ligand percentage identified by mass spectrometry in studies [35,36,37,38] is shown in **C** panel; the CTL epitope percentage from [37] is shown in **D** panel, and the total IFN-γ^+^ immune response percentage detected in the HLA class I-transgenic mice from [37] is shown in **E** panel.

Although no correlation was found between the number and nature of the HLA ligands detected in the immunoproteomics analyses with respect to either HRSV protein size or the content in the residues used by anchor motif amino acids for the different HLA class I alleles analyzed, a striking grouping of viral HLA class I ligands was detected. The HRSV genome is ordered as follows: 3′ NS1-NS2-N-P-M-SH-G-F-M2/M2-2-L, where the genes are transcribed sequentially by the polymerase from the single 3′ promoter using a sequential start-stop-restart mechanism. Thus, the promoter-proximal genes are expressed more efficiently than the promoter-distal genes. Therefore, the viral transcription implies a de facto mRNA synthesis gradient that is inversely proportional to the distance of each HRSV gene from the 3′ end of the genome [21,41,42]. Under this gradient and similarly to other mononegavirales, the HRSV genome has been divided into three different mRNA transcription groups [20]: 3′ core protein genes (Figure 2A, open slices), intermediate (IM) genes (Figure 2A, dotted slices), and the 5′ large polymerase gene (Figure 2A, closed slice). The 3′, IM and 5′ groups involve 26% (NS1, NS2, N, P, and M proteins), 25% (SH, G, F, M2, and M2-2 proteins) and 49% (L protein) of the HRSV proteome, respectively (Figure 2B). Of the 17 physiologically processed HRSV ligands identified by mass spectrometry in different studies, most of them (70%) were included in proteins encoded by the 3′ group, whereas only 24% and 6% of them were derived from proteins encoded by the IM and 5′ groups, respectively (Figure 2C). This 12:4:1 distribution of HLA class I ligands found in the overall tally of the immunoproteomics analysis was significantly different from an expected random distribution along the HRSV proteome. In the expected distribution, the 3′ and IM groups should each be the source of a quarter of the natural ligands, and half of all viral HLA class I-restricted peptides should be derived from the 5′ group L protein. Therefore, the immunoprevalence of the HLA class I response that the proteins to which HLA class I antigen processing and presentation are addressed to is determined by the HRSV transcription groups. Similarly, a ligand from the C protein included in the 3‘ transcription group of the paramyxo-measles virus was the HLA class I immunodominant epitope, and the other three ligands from the F and H proteins, which were included in the intermediate group or M protein of the 3‘ group, were subdominant [43].

## 3. The Immunodominance of the T Cell Class I-Specific Response against HRSV is Limited by the Viral Transcription Group

Different HLA class I (-A*02, -B*07, or -B*27) transgenic mice infected with HRSV were utilized for studying the relevance of the HLA class I viral ligands previously identified by mass spectrometry in vivo [37]. Measurement by Enzyme-Linked ImmunoSpot Assay (ELISPOT) analysis of the functional ex vivo activity of T cells showed that all the HLA-A*02, two of the three HLA-B*07, and six of the nine HLA-B*27 viral ligands previously identified were natural epitopes restricted by their respective HLA class I-presenting molecule and were simultaneously recognized as part of the acute response to HRSV (Figure 2D). 

In each of the three HLA transgenic models utilized, the epitopes derived from viral proteins included in the 3′ group showed higher specific IFN-γ^+^-secreting responses than the corresponding ligands for the IM and 5′ groups. Quantification of the whole T cell responses specific to the 11 epitopes presented by the three HLA class I molecules showed that the vast majority (90%) of the specific IFN-γ^+^ responses (measured by ELISPOT) were restricted by ligands from proteins that were encoded by this 3′ group, with the N protein being the main target (79%) of the cytotoxic immune response (Figure 2E). Thus, the HLA class I-restricted, T cell-specific response hierarchy against HRSV was focused toward the 3′ viral transcription group. 

## 4. HLA Class II Immunoproteomics: The Viral Transcriptional Gradient does not Determine Immunoprevalence or Immunodominance, Which are Mainly Focused on the F Protein

As in the HLA class I-restricted cytotoxic cellular immune response, some HRSV epitopes presented by different HLA class II molecules have been identified using T cells from seropositive individuals. However, as was the case for experiments on class I epitopes, these experiments were not performed against the complete virus but only with overlapping synthetic peptides from a short fragment of 21 residues from the G protein [44] or the F protein [45,46]. To date, only one study with two patients has attempted to determine the full array of HRSV ligands presented by HLA class II molecules [47]. These CD4^+^ T cells, which were restricted by two different HLA-DP alleles, were specific for two different peptides from the matrix and attachment G proteins, respectively [47]. Thus, is the T helper immune response directed against one epitope or, as for the HLA class I response, against various epitopes from HRSV? To answer this question, an immunoproteomic analysis of viral ligands presented by several frequent class II molecules (HLA-DR*04 and HLA-DR*13) that were isolated from HRSV long strain-infected lymphoblastoid cell line was carried out [48]. This study demonstrated the existence of nineteen natural HRSV ligands bound to several HLA class II molecules. Sixteen of these ligands were included in four complex nested sets of peptides, with C- and N-terminal extensions from a minimal core sequence, as is usual in HLA class II antigen processing (Table 3). HLA class II binding was analyzed using bioinformatics tools; these tools predicted that most ligands identified by mass spectrometry could bind up to all four of the different HLA-DR molecules expressed by the cell line utilized (Table 3). This promiscuity of binding could be relatively common in HLA class II antigen presentation [49]. 

**Figure 3:**
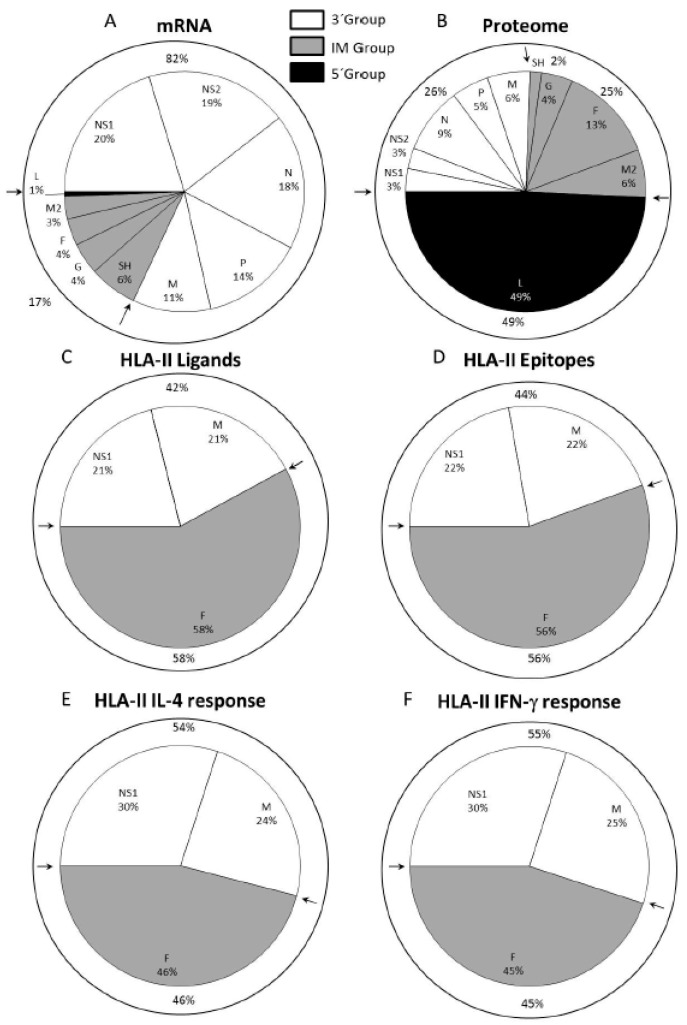
HLA class II ligands from HRSV identified by mass spectrometry and their relation to the viral mRNA, proteome, and T cell helper immune response. Pie chart representation of the HRSV genome indicating the relative abundance of mRNAs due to viral transcription (**A** panel) and the percentage of the proteome (**B** panel) for each protein, as described in Figure 1. For each protein included in its respective transcription group, the HLA class II ligand percentage identified by mass spectrometry in study [48] is shown in **C** panel, and the CD4^+^ epitope percentage from [48] is shown in **D** panel. The total of either IL-4^+^ or IFN-γ^+^ immune helper response percentages that were detected in the HLA class II-transgenic mice in study [48] is shown in the **E** and **F** panels, respectively.

Next, the physiological relevance of each of the HLA class II viral ligands identified by mass spectrometry was tested in vivo in an animal model, specifically, in HLA-DRB1*0404 transgenic mice infected with HRSV. Six of the seven peptides corresponding to the longest ligand of each nested set of the identified viral ligands were simultaneously recognized as part of the acute response to HRSV (Table 3 and Figure 3D). Five of these peptides induced both Th1 and Th2 cytokine expression, which were measured by IFN-γ^+^ and IL-4 secretion, respectively (Table 3), whereas the other peptide (F_91-107_) only induced a Th2 cytokine expression pattern in spleen cells from the HRSV-infected mice (Table 3) [48]. The quantification of the overall T cell responses showed that the specific analyzed IL-4^+^ and IFN-γ^+^ mediated responses in HLA-DRB1*0404 mice were restricted mainly against ligands from the F protein encoded by the intermediate group; however, ligands from the NS1 and M proteins that are encoded by the 3‘ group were also significantly presented and recognized, as shown in Figure 3E,F. 

Thus, collectively, both the immunoprevalence and the immunodominance of the HLA class II response against this virus were focused on one nonstructural (NS1) and two structural (matrix and mainly fusion) proteins of the infective virus, in contrast to the HLA class I response against HRSV, which was overwhelmingly directed toward ligands from proteins encoded by the 3′ group. This dual immune response between HLA class I and II immunoprevalence and immunodominance correlated with the different antigen processing pathways: the peptides bound to the HLA class I molecules are derived mainly from proteolytic processing of newly synthesized viral proteins in the cytosol [50]. By contrast, the HLA class II ligands are generated from viral particles or proteins that are endocytosed and later degraded in endosomes by acid-dependent proteases [50].

## 5. Developing a Vaccine against HRSV

Both the immunoprevalence and the immunodominance of the HLA class I and II cellular responses defined in the immunoproteomics studies could have implications for vaccine development. These responses are focused on proteins from the 3′ core (NS1, N, and M) and intermediate transcriptional (F protein) groups, genes that encode 51% of the HRSV proteome but constitute 94% (Figure 2C) and 100% (Figure 3C) of the natural HLA class I and II ligands identified in these immunoproteomics studies, respectively. Moreover, the epitopes derived from proteins encoded by these two transcriptional groups are responsible for 96% and 100% of the cellular immune responses against HRSV in the HLA transgenic mouse model (Figure 2E and Figure 3E,F). These two transcription groups encoded approximately half of the HRSV Long strain proteome (2237/4402 residues). A recombinant vaccine expressing the four HRSV (NS1, N, M, and F) proteins against which both the HLA class I and II immune responses are mainly targeted represents only 31% of viral proteome (1358 residues); however, nearly every aspect of the antiviral T cell responses against HRSV is preserved by these four viral proteins: 92% (Figure 2E) and 100% (Figure 3E,F) of HLA class I and II-restricted responses, respectively.

An important issue for HRSV vaccine development relates to the NS1 protein, which is a small protein of 139 amino acids. This protein, together with the other non-structural protein (NS2) of this pneumovirus, affects multiple cellular signaling proteins, interfering with both the induction and the cytotoxic functions of type I and III IFNs in human epithelial cells and in immune system cells such as macrophages or dendritic cells, suppressing the innate immune response [51,52]. Thus, their exclusion from any therapeutic immunogen would be reasonable and desirable. The use of only the N, M, and F proteins would not significantly affect a future vaccine because this structure will preserve most of the immunological properties: 89% and 70% of HLA class I- and HLA class II-restricted immune responses, respectively. 

Another relevant point to consider in the vaccine design against HRSV is that this virus shows two different antigenic subgroups: A and B [1]. Within each subgroup, the percentage of nucleotide and amino acid identity between different viral isolates is significantly higher for all the proteins: 97–100% amino acid identity. Between subgroups, most HRSV proteins are also highly conserved (88–96% amino acids identity). However, greater differences are observed for the M2-2, G and SH proteins, which show only 61–71% amino acid identity between subgroups. Thus, it would be reasonable to infer a high percentage of identity of the HLA class I- and II-restricted ligands between the HRSV subgroup strains. Nevertheless, a sequence comparison analysis between the HLA ligands from Long and the other A strains showed that a significant number of them were mutated in at least one residue (6 of 24, 25% in Table 4). Furthermore, 17 of these ligands (71%) are also altered in their sequence in subgroup B strains in relation to the Long strain utilized in the mass immunoproteomics analyses (Table 4). In addition to evidence that CD8^+^ T cells of the respiratory tract are in part functionally inactivated in HRSV-infected mice [53], the surface expression of non-conserved HRSV ligands could partially explain the mechanism of reinfection with different HRSV strains from the same or different antigenic subgroups [54]. Thus, these data have evident implications for antiviral vaccine design. To limit the loss of cross-reactivity between different strains, the HRSV vaccine construct must encode the relevant proteins; that is, the vaccine must encode those amino acid sequences identified by the immunoproteomics analysis in almost one virus strain that is representative of each HRSV antigenic subgroup. For example, the inclusion of Long and 9320 strains in the same vaccine allows the presentation of 16 of 24 ligands detected by mass spectrometry from all viral strains (Table 3).

In summary, based on immunoproteomics studies and different properties of HRSV, a vaccine construct encoding the N, M, and the F proteins from two different HRSV strains (e.g., long and 9320) could be a promising vaccine against this virus that could stimulate both helper and cytotoxic cellular immune responses. In addition, this therapeutic immunogen would have the additional advantage of stimulating the humoral immune response against HRSV because it is well known that the neutralizing mAbs against the HRSV F protein have demonstrated a protective effect of prophylactic serum HRSV-neutralizing antibodies against severe HRSV disease [55,56]. 

## 6. Conclusions

HRSV lacks an effective approved vaccine or preventive antiviral therapy, and currently, the management of infected patients (mainly infants) is purely supportive. One HRSV monoclonal antibody is available as prophylaxis against severe infection, but it is used only in a tiny proportion of infants. Worldwide, this virus remains one of the pathogens deemed most important for vaccine development [57], as is shown by the fact that in the last 50 years several efforts have been made toward HRSV vaccine design using different experimental approaches (reviewed in [19]). However, these efforts primarily are focused on the F protein. Usually, the viral envelope proteins are good targets for triggering efficient humoral immune responses, but their contributions to both cytotoxic and helper cellular immune responses are unclear. Ideally, a vaccine must elicit a strong memory humoral and cellular immune response. In recent years, different immunoproteomics studies have delineated the diversity of naturally processed ligands and epitopes against both the helper and cytotoxic cellular immune response that is addressed to several common HLA class I and II molecules. In these studies, both the immunoprevalence and the immunodominance of HLA class I-restricted effector response was directed against peptides from viral proteins encoded by the 3′ transcription group of HRSV, mainly nucleoprotein and matrix protein. In contrast, the ligands and epitopes from HLA class II-restricted helper response against this virus were mostly derived from fusion and matrix proteins. In summary, the picture emerging from these studies is that the cellular immune response against HRSV is mainly focused on three viral proteins: nucleoprotein, matrix and fusion. Since the HRSV fusion protein is the main target of the neutralizing antibody response, a vaccine including these viral proteins could efficiently stimulate the three major components of the adaptive immune system: humoral, helper and effector cellular immune responses.

## Figures and Tables

**Table 1:** Summary of human leukocyte antigen (HLA) class I epitopes of human respiratory syncytial virus (HRSV) identified using cytotoxic T lymphocytes (CTL) from seropositive individuals.

Protein	Position	Sequence	HLA-I	Reference
Fusion (F)	109–118	RELPRFMNYT	HLA-A*01	[31]
Matrix (M)	229–237	YLEKESIYY	HLA-A*01	[39]
Nucleoprotein (N)	16–24	QLLSSSKYT	HLA-A*02	[34]
Nucleoprotein (N)	137–145	KMLKEMGEV	HLA-A*02	[34]
Nucleoprotein (N)	303–311	ILNNPKASL	HLA-A*02	[34]
Matrix 2 (M2)	151–159	RLPADVLKK	HLA-A*03	[39]
Nucleoprotein (N)	308–316	NPKASLLSL	HLA-B*07	[34]
Nucleoprotein (N)	258–266	VMLRWGVLA	HLA-B*08	[32]
Matrix 2 (M2)	64–72	AELDRTEEY	HLA-B*44	[39]
Matrix (M)	195–203	IPYSGLLLV	HLA-B*51	[39]
Non-structural protein 2 (NS2)	41–49	LAKAVIHTI	HLA-B*51	[39]
Fusion (F)	118–126	RARRELPRF	HLA-B*57	[30]
Fusion (F)	551–559	IAVGLLLYC	HLA-C*12	[30]

**Table 2:** Summary of the viral HLA class I natural ligands identified by immunoproteomics analyses in HRSV-infected cells.

Protein ^a^	Position	Transcription Group ^b^	Sequence	HLA-I	IFN-γ^+^ Response	Reference
NS1	33–41	3′	KLIHLTNAL	HLA-A*02	+ ^c^	[35,37]
N	315–323	3′	TQFPHFSSV	HLA-A*02	+	[37]
F	229–239	IM	RLLEITREFSV	HLA-A*02	+	[37]
NS2	19–30	3′	RPLSLETTITSL	HLA-B*07	+	[37]
N	306–314	3′	NPKASLLSL	HLA-B*07	+	[37]
F	551–559	IM	KARSTPVTL	HLA-B*07	ND ^d^	[35]
NS2	37–45	3′	HRFIYLINH	HLA-B*27	-	[36]
N	100–109	3′	HRQDINGKEM	HLA-B*27	+	[36]
N	184–194	3′	RRANNVLKNEM	HLA-B*27	+	[36]
N	195–205	3′	KRYKGLLPKDI	HLA-B*27	+	[36]
P	198–208	3′	LRNEESEKMAK	HLA-B*27	-	[36]
M	76–84	3′	SRSALLAQM	HLA-B*27	-	[36]
M	169–177	3′	VRNKDLNTL	HLA-B*27	+	[36]
M2	150–159	IM	KRLPADVLKK	HLA-B*27	+	[36]
L	2089–2097	5′	GRNEVFSNK	HLA-B*27	+	[36]
M	188–198	3′	AITNAKII	HLA-C*04	ND	[38]
G	25–33	IM	FISSGLYKL	HLA-C*07	ND	[37]

^a^ The abbreviations used for viral proteins were NS1 (non-structural protein 1), NS2 (non-structural protein 2], N (nucleoprotein), P (phosphoprotein), M (matrix protein), SH (small hydrophobic protein), G (glycoprotein), F (fusion protein), M2-2 (matrix protein 2], and L (polymerase). ^b^ The proteins were grouped into three different transcription groups: 3′, IM, and 5′ as in previously publications [21,41,42]. ^c^ Response in the respective HLA class I transgenic mouse model infected with HRSV from [37]. ^d^ ND: not determined.

**Table 3:** Summary of the viral HLA-DR natural ligands identified by immunoproteomics analyses in HRSV-infected cells.

Protein	Position	Sequence	HLA-DR Theoretical Binding				Response ^a^	
			B1*0404	B4*0101	B1*1301	B3*0201	IL-4^+^	IFN-γ^+^
NS1	31–44	TDKLIHLTNALAKA	+ ^b^	+	+	+	+	+
NS1	31–46	TDKLIHLTNALAKAVI	+	+	+	+	ND ^c^	ND
NS1	31–47	TDKLIHLTNALAKAVIH	+	+	+	+	ND	ND
NS1	30–47	YTDKLIHLTNALAKAVIH	+	+	+	+	+	+
M	57–69	ILVKQISTPKGPS	+	+	+	+	+	+
M	55–69	NILVKQISTPKGPS	+	+	+	+	ND	ND
M	55–70	ILVKQISTPKGPSL	+	+	+	+	ND	ND
M	55–71	VNILVKQISTPKGPSLR	+	+	+	+	+	+
F	96–107	LMQSTPAANNRA	+	+	+	+	-	+
F	92–106	ELQLLMQSTPAANNR	+	+	+	+	ND	ND
F	92–107	ELQLLMQSTPAANNRA	+	+	+	+	ND	ND
F	91–107	TELQLLMQSTPAANNRA	+	+	+	+	-	+
F	176–187	KAVVSLSNGVSV	+	+	+	+	+	+
F	174–189	TNKAVVSLSNGVSVLT	+	+	+	+	ND	ND
F	174–190	NKAVVSLSNGVSVLTS	+	+	+	+	ND	ND
F	173–196	STNKAVVSLSNGVSVLTSKVLDLK	+	+	+	+	+	+
F	199–215	IDKQLLPIVNKQSCRIS	+	+	+	+	+	+
F	400–420	TDVSSSVITSLGAIVSCYGKT	+	-	+	+	-	-
F	428–441	NRGIIKTFSNGCDY	+	-	-	-	+	+

^a^ Response in HLA-DRB1*0404 transgenic mice infected with HRSV from [48]. ^b^ Theoretical binding to HLA-DR as calculated in the NetMHCIIpan-3.0 server from [48]. ^c^ ND: not done.

**Table 4:** Conservation of HLA viral ligands in several HRSV strains.

**Strain ^a^**	**S1 _30–47_** ^b^ (A*02^c^, DR*04)		N _315–323_ (A*02)	F _229–239_ (A*02)	NS2 _19–30_ (B*07)	N _306–314_ (B*07)	F _551–559_ (B*07)	M _188–195_ (C*04)	G _25–33_ (C*07)
Long	YTDKLIHLTNALAKAVIH		TQFPHFSSV	RLLEITREFSV	RPLSLETTITSL	NPKASLLSL	KARSTPVTL	AITNAKII	FISSGLYKL
S2	------------------		---------	-----------	-------I-I--	---------	---------	--------	----C----
A2	------------------		---------	-----------	-------I----	---------	---------	--------	----C----
9320	------L--------A--		----N----	-----------	----M-SI----	---------	--KN-----	--------	V---C--R-
18537	------L-----------		----N----	-----------	----M-SI----	---------	--KN-----	ND	V---C--R-
B1	------L--------A--		----N----	-----N-----	----MDSI----	---------	--KN-----	--------	V---C--R-
**Strain**	NS2 _37–45_ (B*27)	N _100–109_ (B*27)	N _184–194_ (B*27)	N _195–205_ (B*27)	P _198–208_ (B*27)	M _76–84_ (B*27)	M _169–177_ (B*27)	M2-22k _150–159_ (B*27)	L _2089–2097_ (B*27)
Long	HRFIYLINH	HRQDINGKEM	RRANNVLKNEM	KRYKGLLPKDI	LRNEESEKMAK	SRSALLAQM	VRNKDLNTL	KRLPADVLKK	GRNEVFSNK
S2	---------	----------	-----------	-----------	-----------	----V----	---------	----------	---------
A2	-K-------	----------	-----------	-----------	-----------	----V----	---------	----------	---------
9320	-K------N	Y---------	----------I	------I----	-----------	----V----	-K-----S-	----------	---------
18537	-K------N	Y---------	----------I	------I----	-----------	ND	ND	----------	---------
B1	-K------N	Y---------	----------I	------I----	-----------	----V----	-K-----S-	----------	---------
**Strain**	M _55-71_ (DR*04)		F _91–107_ (DR*04)	F _173–196_ (DR*04)		F _199–215_ (DR*04)	F _400–420_ (DR*04)		F _428–441_ (DR*04)
Long	VNILVKQISTPKGPSLR		TELQLLMQSTPAANNRA	STNKAVVSLSNGVSVLTSKVLDLK		IDKQLLPIVNKQSCRIS	TDVSSSVITSLGAIVSCYGKT		NRGIIKTFSNGCDY
S2	-----------------		------------T----	------------------------		--------------S--	---------------------		--------------
A2	-----------------		-----------PT----	------------------------		--------------S--	---------------------		--------------
9320	I----------------		------T-N--------	------------------------		-NN-------Q------	--I------------------		--------------
18537	I----------------		--------N--------	------------------------		-NNR------Q------	--I------------------		--------------
B1	-----------------		--------N--------	------------------------		-NN-------Q------	--I------------------		--------------

**^a^** Long, S2 and A2 strains are representative of HRSV subtype A, whereas 9320, 18537 and B1 are HRSV B strains. **^b^** Protein and position of each ligand and in parenthesis the respective HLA presenting molecule. **^c^** The NS1 _33–41_ ligand included into NS1 _30-47_ sequence is showed in red.
